# Airway epithelial cell–lung macrophage crosstalk in type 2-high and low asthma endotypes: mechanisms and therapeutic strategies

**DOI:** 10.3389/fimmu.2026.1985258

**Published:** 2026-10-22

**Authors:** Yinuo Wang, Yuxuan Sun, Suning Mu, Yan Wang, Chaofan Guo, Youpeng Wang

**Affiliations:** 1 Faculty of Clinical Medical College, Heilongjiang University of Chinese Medicine, Harbin, Heilongjiang, China; 2 Institute of Basic Theory of Chinese Medicine, China Academy of Chinese Medical Sciences, Beijing, China; 3 Department of Pediatrics, The Second Affiliated Hospital of Heilongjiang University of Chinese Medicine, Harbin, Heilongjiang, China

**Keywords:** airway epithelial cells, airway inflammation, asthma, crosstalk, lung macrophages, type 2-high asthma, type 2-low asthma

## Abstract

Asthma is a heterogeneous chronic airway disease. Asthma endotypes are mainly classified as type 2-high and type 2-low, based on immunological and molecular mechanisms, providing a more comprehensive framework for understanding the underlying pathological processes. Airway epithelial cells (AECs) and lung macrophages contribute to airway homeostasis and asthmatic inflammation through communication that can support either tissue protection or disease-associated responses. This narrative review integrates their physiological and pathological functions, focusing on AEC-to-macrophage and macrophage-to-AEC signaling within broader multicellular networks across different asthma endotypes. Direct functional evidence for intercellular communication from associations and indirect mechanisms is distinguished and graded. Therapeutic strategies targeting AECs, lung macrophages, and their crosstalk, including both clinically validated and preclinical approaches, are also discussed. The AEC–macrophage axis exhibits context-dependent signaling that may amplify inflammation or support protective regulation, providing a basis for evaluating potential therapeutic interventions.

## 1 Introduction

Asthma affects more than 300 million people worldwide, and its prevalence continues to increase globally (1). According to the latest Global Initiative for Asthma report, asthma is defined as a heterogeneous chronic airway disease characterized by airway inflammation, airway hyperresponsiveness (AHR), and airway remodeling (2). Because the traditional allergic/nonallergic classification does not adequately reflect its mechanistic and therapeutic heterogeneity, asthma is increasingly categorized into type 2-high and type 2-low endotypes based on the predominant inflammatory pathways (3).

Type 2-high asthma is mainly driven by type 2 immune responses mediated by T helper 2 (Th2) cells and group 2 innate lymphoid cells (ILC2s). It is characterized by the production of type 2 cytokines, including interleukin (IL)-4, IL-5, and IL-13, and is typically associated with eosinophilic airway inflammation, increased fractional exhaled nitric oxide (FeNO) and immunoglobulin(Ig) E levels (4). In contrast, type 2-low asthma is a more heterogeneous endotype lacking well-defined biomarkers, typically with lower eosinophil counts and FeNO, and is often associated with neutrophilic inflammation, corticosteroid insensitivity, infection, obesity-related inflammation, and severe asthma (5, 6).

Airway epithelial cells (AECs) form the first physical and immunological barrier between the respiratory tract and the external environment, and are key regulators of airway inflammation in asthma (7). In type 2-high asthma, AECs initiate and exacerbate type 2 inflammation by releasing alarmins and chemokines (8). Whereas in type 2-low asthma, epithelial activation of innate immune pathways promotes non-type 2 inflammatory responses (5). Lung macrophages are also central to airway immunity and exhibit distinct functional and polarization states across asthma endotypes.

Under physiological conditions, AECs and lung macrophages communicate bidirectionally to maintain airway immune homeostasis. AECs regulate macrophage tolerance and host defense, whereas macrophages clear apoptotic and damaged cells and promote epithelial repair (9, 10). In asthma, dysregulation of both AECs and lung macrophages can establish pathological crosstalk that contributes to airway inflammation and remodeling. Although previous studies have characterized the independent roles of AECs (11) and macrophages (12) in asthma pathogenesis, the mechanisms underlying their bidirectional interactions remain incompletely understood.

This review organizes the evidence by the interaction direction of AEC-macrophage signaling and the asthma inflammatory endotype, distinguishing type 2-high mechanisms and findings relevant to type 2-low asthma. We consider cellular sources and recipients, distinguish and grade the direct functional findings from associative or indirect evidence, and place these interactions within broader multicellular networks. This review further links the balance between pathogenic and protective communication to therapeutic strategies while separating clinical validation of mediator targeting from preclinical evidence for selective modulation of the AEC–macrophage axis, thereby providing valuable insights and targets for mechanistic research and clinical treatment of asthma.

## 2 Literature search and selection strategy

This article is a narrative review that summarizes recent advances in AEC–lung macrophage crosstalk across different asthma endotypes and discusses their potential therapeutic implications. A literature search was conducted in PubMed, Web of Science, Scopus, Google Scholar, and the Cochrane Library for articles published up to August 2026. The search strategy included combinations of keywords related to airway epithelial cells, macrophages, monocyte-derived macrophages, lung macrophages, alveolar/interstitial/airway macrophages, asthma, type 2 inflammation, type 2-high asthma, type 2-low asthma, cell–cell communication, crosstalk, extracellular vesicles, exosomes, and therapy. Relevant original research articles, clinical studies, and review articles were considered based on their relevance to the scope of this review. Additionally, the reference lists of selected articles were manually reviewed.

## 3 AECs in asthma

### 3.1 Physiological function of AECs

Airway epithelial lineages originate from respiratory epithelial progenitors specified from the ventral anterior foregut endoderm (13). In mature conducting airways, AECs form a pseudostratified epithelium composed mainly of basal, ciliated, goblet, and club cells, together with rare cell types such as ionocytes, neuroendocrine cells, and tuft cells (14). Ciliated cells mediate mucociliary clearance through the coordinated beating of motile cilia (15). Goblet cells primarily produce mucins that form the mucus barrier, and club cells secrete secretoglobins and other host-defense molecules, contributing to immune defense, xenobiotic metabolism, and epithelial repair (16). Basal cells are the principal progenitor cells that replenish ciliated, secretory, and selected rare epithelial cell types after injury (17).

The physical barrier function of the airway epithelium depends on the integrity, polarity, and junctional complexes of AECs. Tight junctions regulate paracellular permeability (18), whereas adherens junctions provide mechanical adhesion and structural stability (19). AECs also support the immune barrier by secreting mucus and antimicrobial and host-defense proteins, and by transporting IgA and IgM from the basolateral membrane to the apical surface to clear or exclude allergens, pathogens, and pollutants (20–22). In addition, epithelial pattern-recognition receptors (PRRs) detect pathogen-associated molecular patterns (PAMPs) and damage-associated molecular patterns (DAMPs), thereby coordinating inflammatory responses and maintaining airway immune homeostasis (7, 23, 24).

### 3.2 The pathophysiology of AECs in asthma

Asthma-associated stimuli, including allergens and pathogens, can directly or indirectly disrupt epithelial junctions, increase barrier permeability, and promote epithelial injury, facilitating antigen and PAMP penetration into the submucosa. Damaged AECs release DAMPs that trigger local innate and adaptive immune activation (23, 25). Inflammatory cytokines further impair barrier integrity (26). Epithelial repair is also defective in asthma, with impaired basal cell differentiation and proliferation and defective wound healing (27). These abnormalities sustain a self-reinforcing cycle of epithelial injury, barrier dysfunction, abnormal repair, and chronic airway inflammation.

#### 3.2.1 AECs in type 2-high asthma

In type 2-high asthma, AECs undergo dynamic pathological changes after allergen exposure. Within 24 h after allergen challenge, epithelial and submucosal IL-33, IL-25, and thymic stromal lymphopoietin (TSLP) levels increase in patients with asthma, with greater responses associated with late-phase airway obstruction (28). House dust mite (HDM), a major aeroallergen, induces alarmin responses in AECs partly through Toll-like receptor (TLR) 4 signaling (29). IL-33 is rapidly released following epithelial injury and reaches high levels within approximately 2 h (30). It promotes IL-5 and IL-13 production by ILC2s while modulating mast cell phenotype and infiltration in asthma (27). Its receptor ST2 is encoded by IL1RL1, and asthma-associated variants in IL-33 and IL1RL1 influence IL-33 expression, asthma susceptibility, and airway type 2 inflammation (31, 32). IL-25 signals through the IL-17RA/IL-17RB complex to activate ILC2s, enhance type 2 immune responses, sustain eosinophilic inflammation, and promote VEGF-associated angiogenic responses in asthma (33, 34). TSLP is also rapidly induced after HDM exposure; its mRNA levels increase within 3 h, and TSLP protein secretion peaks at 6 h (35). TSLP promotes type 2 immunity by activating dendritic cells (DCs) (36), mast cells (37), and eosinophils (38), while potentially impairing antiviral IgA responses (39).

AEC-derived alarmins activate DCs and ILC2s, promoting Th2 polarization and type 2 inflammation (40–43). ILC2s and Th2 cells subsequently produce IL-4, IL-5, and IL-13 (3, 44). IL-4 promotes Th2 differentiation and IgE class switching and, together with IL-13, impairs epithelial barrier integrity (45). IL-5 supports eosinophil maturation, survival, and airway recruitment (46). IL-13 acts directly on AECs to induce goblet-cell metaplasia and mucus overproduction (47), suppress ciliated cell differentiation and ciliary function, impair mucociliary clearance, and disrupt tight junctions and increase epithelial permeability (48, 49). During the chronic phase of type 2-high asthma, persistent AEC dysfunction contributes to airway remodeling. In HDM models, airway inflammation and goblet-cell hyperplasia emerge after 2 weeks of exposure, while subepithelial collagen deposition remains limited; by 4 weeks, airway inflammation, allergic response, and collagen deposition become more prominent, reaching remodeling features comparable to those seen after 8 weeks (50, 51). Chronic IL-13 stimulation similarly drives pan-epithelial remodeling in human airway epithelial cultures (52), while sustained IL-4/IL-13 exposure can imprint allergic inflammatory memory in airway basal progenitor cells, reducing epithelial differentiation diversity and impairing barrier function (53).

AEC-derived alarmins also contribute to early airway remodeling. IL-25 promotes goblet-cell hyperplasia and mucus hypersecretion (54, 55); it also contributes to allergic airway remodeling and increases IL-33 and TSLP production (56), whereas IL-33 and TSLP are associated with mucus metaplasia and plugging (27, 57). TSLP may additionally promote epithelial-mesenchymal transition (EMT) through transforming growth factor (TGF)-β1 (58), and enhance airway smooth muscle cell (ASMC) proliferation and migration (59, 60).

Overall, in early type 2-high asthma, AEC-derived alarmins activate DCs and ILC2s, promote Th2 polarization, initiate type 2 inflammation, and may also contribute to early airway remodeling. As inflammation persists, type 2 cytokines act back on AECs to establish a self-reinforcing inflammatory circuit. In chronic phases, dysregulated AECs further exacerbate progressive airway remodeling.

#### 3.2.2 AECs in type 2-low asthma

Type 2-low asthma is highly heterogeneous and lacks well-validated biomarkers. IL-17-related inflammatory programs have been implicated in subsets of severe, neutrophilic, and corticosteroid-insensitive asthma (5, 61). In an HDM/lipopolysaccharide (LPS)-induced neutrophilic asthma model, increased bronchial epithelial IL-17A expression was accompanied by neutrophil infiltration, AHR, goblet-cell metaplasia, and mucus accumulation (62). In human bronchial epithelial cells, IL-17A together with tumor necrosis factor (TNF)-α induced an epithelial gene signature comprising CXCL1, CXCL2, CXCL3, IL8, and CSF3 (63). This IL-17-inducible signature was also identified in severe asthma and linked to interactions between activated T cells and AECs, potentially contributing to neutrophilic and corticosteroid-insensitive inflammation (61). AECs can additionally release IL-17C after infectious or inflammatory stimulation, promoting type 17 inflammation through IL-17C-responsive immune cells (64, 65). Conversely, AECs themselves are important effectors of IL-17 signaling. Combined IL-17A/F and TNF-α stimulation increases epithelial production of GROα, IL-8, monocyte chemoattractant protein-1 (MCP-1), and elafin, supporting a non-type 2 epithelial state characterized by neutrophil recruitment and host-defense responses (66).

IL-6 signaling represents another AEC-related inflammatory program relevant to type 2-low asthma (5). Elevated IL-6 levels have been associated with severe asthma, obesity-related asthma, peripheral blood neutrophilia, impaired lung function, and systemic corticosteroid use (67, 68). In human bronchial epithelial air-liquid interface cultures, an IL-6 trans-signaling signature defined an asthma phenotype with limited type 2 inflammation but greater overall airway inflammation, frequent exacerbations, submucosal T-cell and macrophage infiltration, increased TLR-pathway gene expression, and reduced expression of cell-junction genes (69). Soluble IL-6 receptor α (sIL-6Rα) further enhanced epithelial IL-6 signaling, and the corresponding epithelial IL-6+sIL-6Rα signature was enriched in bronchial biopsies from patients with asthma and associated with lower sputum eosinophil counts, supporting a potential role for epithelial IL-6 signaling in non-eosinophilic airway inflammation in type 2-low asthma (70).

Beyond these pathways, bronchial epithelial cells from patients with asthma exhibit deficient interferon (IFN)-β or IFN-λ responses to rhinovirus, accompanied by increased viral replication, epithelial injury, and exacerbation severity (71, 72). Conversely, some patients with neutrophilic asthma show increased airway IFN-β, IFN-λ1/IL-29, and interferon-stimulated gene expression (73, 74), suggesting that antiviral IFN responses in AECs may be either insufficient at an early stage or persistently activated, with the latter potentially exacerbating non-type 2 airway inflammation. In specific contexts, TSLP can promote Th17 differentiation and IL-17A/IL-17F responses (75). IL-33/ST2 signaling can activate neutrophils and macrophages and has been linked to neutrophilic airway inflammation and uncontrolled asthma (76). In summary, although the pathobiology of type 2-low asthma is highly heterogeneous, AECs are key mediators between exogenous stimuli and non-type 2 airway inflammation. Epithelial IL-17- and IL-6-related programs, infection-associated responses, and other AEC-derived mediators can contribute to type 2-low asthma.

## 4 Lung macrophages in asthma

### 4.1 Physiological function of lung macrophages

Lung macrophages consist primarily of alveolar macrophages (AMs) and interstitial macrophages (IMs) (77). Airway macrophages residing at or near the epithelial surface are increasingly recognized as an additional lung macrophage population, although their definition remains unsettled and they were historically subsumed as part of the AMs (9, 78). Recent studies identify intraepithelial, submucosal, and tracheal subsets, some of which are replenished by circulating monocytes after injury, indicating that they are not simply extensions of AMs (79, 80). AMs reside on the alveolar epithelial surface and constitute the predominant resident macrophage population in the healthy alveolar space (81). They originate from fetal monocytes and are maintained mainly through local proliferation, with limited contribution from circulating monocytes (82, 83). AMs express markers including CD64, CD11c, CD169 and MerTK (84). They clear inhaled particles, pathogens, and cellular debris through phagocytosis, participate in surfactant uptake and metabolism, and regulate antimicrobial defense and epithelial integrity, thereby supporting alveolar homeostasis (84–86). IMs reside in the lung interstitium and derive from both fetal precursors and circulating monocytes (87). They are heterogeneous and can be distinguished by markers including CD206, MHC II, CX3CR1, LYVE1, and CCR2 (88, 89). IMs can be further divided into different subsets, and classification schemes vary across studies (89, 90). Positioned close to airway structures, IMs regulate the local immune microenvironment and participate in antigen presentation, regulation of local immunity and tolerance, tissue repair, and fibrosis. However, unlike AMs, IMs lack the capacity to support pulmonary surfactant production and maintenance (84, 86).

### 4.2 The pathophysiology of lung macrophages in asthma

Macrophages are key innate immune cells in asthma (12). Allergen exposure and epithelial injury induce monocyte-recruiting chemokines, promoting the influx of circulating monocytes into the airways and their differentiation into recruited macrophages (77, 91). Across asthma endotypes and disease stages, lung macrophages, including resident and monocyte-derived macrophages, exhibit changes in polarization and functional states (81). The conventional M1/M2 polarization framework distinguishes classically activated M1 macrophages, typically induced by lipopolysaccharide (LPS) and IFN-γ and associated with pro-inflammatory/injury responses, from selectively activated M2 macrophages, which are generally linked to anti-inflammatory/repair processes. M2 macrophages have been subdivided into M2a, M2b, M2c, and M2d phenotypes, induced mainly by IL-4/IL-13, immune complexes combined with LPS or IL-1R signaling, IL-10/TGF-β/glucocorticoids, and LPS/IL-6/A2R ligands, respectively (92–94). However, macrophage activation in asthma spans a continuum of overlapping functional states rather than fixed M1/M2 phenotypes (95); this review uses the simplified M1/M2 framework for ease of understanding.

#### 4.2.1 Lung macrophages in type 2-high asthma

In type 2-high asthma, AEC-derived alarmins promote type 2 cytokine production, including IL-4 and IL-13 (24). AECs also produce C-C motif chemokine ligand 2 (CCL2), which recruits circulating monocytes into the airway and lung tissue (91). Draijer et al. reported that during early HDM exposure, non-polarized resident macrophages transiently expanded, with AMs maintained mainly by local proliferation and circulating monocytes contributing predominantly to IM replenishment, while repeated HDM exposure for 2 weeks promoted M2-like lung macrophage activation (96). Responses to type 2 cytokines vary among lung macrophage populations; the lung microenvironment can constrain IL-4 responsiveness in resident AMs, whereas IMs show more pronounced activation during type 2 inflammation (97). M2-associated markers also differ between species. In mice, Arg1, Ym1, and Fizz1 are commonly upregulated (98, 99), whereas human asthma is more often associated with increased CCL13, CCL17, CCL18, CCL22, and CD206 expression (93).

The increased abundance of M2-like macrophages exacerbates eosinophilic inflammation and airway remodeling in allergic asthma (100). Winkler et al. reported in an ovalbumin (OVA)-induced allergic asthma mouse model that IL-4 and IL-13 levels in bronchoalveolar lavage fluid (BALF) peaked 24 h after the final allergen challenge, whereas eosinophilia persisted for at least 1 week, and M2-like markers in AMs were expressed at higher levels than in the control group even 3 weeks later. These proinflammatory macrophages promoted eosinophil recruitment, participating in the persistence of type 2 inflammation after acute allergen exposure (99). Draijer et al. indicated that suppressing Ym1^+^ M2 macrophages reduced eosinophilic inflammation and collagen deposition in mice, but increased neutrophilic inflammation and aggravated AHR (100), highlighting their context-dependent functions.

During injury resolution, M2-like macrophages support damaged tissue clearance, extracellular matrix (ECM) remodeling, and epithelial repair through TGF-β, platelet-derived growth factor (PDGF), and other repair mediators (12). However, persistent allergen exposure and sustained type 2 inflammation can redirect these repair programs toward pathological remodeling. Henkel et al. found that HDM exposure accompanied by sustained type 2 inflammation reprogrammed macrophage lipid metabolism, increasing cysteinyl leukotrienes and 12-/15-lipoxygenase metabolites (101). M2-associated Arg1 can reduce NO production by competing for L-arginine, subsequently promoting ornithine, polyamine, and proline metabolism, thereby contributing to AHR, hyperplasia, and collagen deposition (102). Ym1 and Fizz1 have likewise been implicated in eosinophil recruitment and fibrotic remodeling, exacerbating airway remodeling (103, 104).

Lechner et al. found that lung macrophages differentiated from monocyte progenitors of HDM-allergic mice or asthma patients exhibited inflammatory transcriptional reprogramming and exaggerated mediator responses upon stimulation (105). They further indicated that macrophages from HDM-allergic mice acquired TNF-dependent inflammatory memory, shifting from an initial type 2 imprint toward classical inflammatory training over time, while *in vitro* HDM exposure generated a distinct CCL17^high^ M2-like state with sustained inflammatory effector functions, perpetuating and exacerbating chronic type 2 airway inflammation (105). Thus, M2-like lung macrophages in type 2-high asthma participate in both inflammatory restraint and tissue repair, but persistent type 2 inflammation can exacerbate eosinophilic inflammation, macrophage metabolic reprogramming, inflammatory memory, and pathological airway remodeling.

#### 4.2.2 Lung macrophages in type 2-low asthma

Lung macrophages contribute to airway host defense through pathogen recognition and phagocytic clearance (77). However, in severe asthma, AMs and monocyte-derived macrophages show impaired phagocytosis of *Haemophilus influenzae* and *Staphylococcus aureus*, a defect that may contribute to bacterial persistence, more frequent exacerbations, and persistent airway inflammation (106). Conversely, specific IM subsets can restrain neutrophilic inflammation. Kawano et al. found in an HDM mouse model that IL-10-producing IMs suppressed Th2/Th17-associated cytokine responses, goblet-cell mucus production, and airway neutrophilia (107). Han et al. identified a CD39^+^CD9^+^ IM subset that similarly limited ATP-dependent neutrophil extracellular trap (NET) formation, Th17 activation, and neutrophilic inflammation through CD9-dependent neutrophil interactions and CD39-mediated ATP degradation. This protective population was suppressed by IL-23 (108).

Macrophage activation states also differ across inflammatory endotypes. Allergic HDM exposure shows prominent type 2 inflammation and M2-dominant macrophages, whereas nonallergic farm dust exposure exhibits neutrophil infiltration, enhanced Th1/Th17 responses, and M1-dominant macrophages (109). In severe human asthma, M1-like macrophage features have been associated with poor corticosteroid responsiveness, while experimental studies support their capacity to promote Th1-associated inflammation and AHR (110). M1-like macrophages typically express CD80, CD86, MHC II, TLR4 and iNOS, and produce pro-inflammatory mediators, including IL-1β, IL-17, IL-6, IL-12, and chemokines. These responses support intracellular pathogen clearance but may also sustain airway inflammation (12, 111).

The NLRP3 inflammasome represents an important macrophage-associated pathway in neutrophilic asthma. Sputum macrophages from patients with neutrophilic asthma show increased expression of NLRP3, caspase-1, and IL-1β (112), while experimental steroid-resistant asthma models indicate that NLRP3/caspase-1/IL-1β signaling promotes neutrophilic inflammation and AHR (113). Persistent nontypeable *Haemophilus influenzae* (NTHi) infection of pulmonary macrophages can exacerbate chronic airway inflammation and Th17 responses, characterized by increased levels of IL-1β, CXCL8, IL-23, and CCL20, in severe neutrophilic asthma, which can further lead to decreased lung function and reduced steroid responsiveness (114). In experimental models, Essilfie et al. reported that lung macrophages produced IL-17 after NTHi infection, followed by sustained neutrophil recruitment (115). They further showed that combining NTHi infection with allergic airway disease promoted the persistence of chronic infection with type 2-low inflammatory features (116). During chronic asthma phases, M1-like macrophage-derived mediators, such as IL-6, may not only exacerbate inflammation but also cooperate with TGF-β-related fibroblast activation to promote subepithelial fibrosis and airway remodeling (111, 117).

Macrophage immunometabolic reprogramming may also sustain type 2-low inflammation (111, 118). M1-like activation is commonly associated with increased glycolysis and decreased tricarboxylic acid cycle activity, providing metabolic support for rapid inflammation and sustained mediator production (119). In mechanistic studies, glycolytic reprogramming promoted succinate accumulation, HIF-1α stabilization, reactive oxygen species (ROS) production, and IL-1β expression (120). RNA sequencing of sputum macrophages from patients with neutrophilic asthma identified transcriptional reprogramming enriched for bacterial responses, neutrophil recruitment, and regulation of phagocytosis/efferocytosis. This signature was associated with poorer lung function and asthma control and greater inhaled corticosteroid use (121).

Overall, lung macrophage dysfunction in type 2-low asthma is heterogeneous and includes both impaired protective effects and acquisition of pro-inflammatory functions. Defective pathogen clearance, Th17/Th1-associated signaling, inflammasome activation, and immunometabolic reprogramming may sustain chronic inflammation in type 2-low asthma. During the chronic phase, these programs may cooperate with other cytokine networks to promote airway remodeling.

## 5 Crosstalk between AECs and lung macrophages in asthma

AEC–lung macrophage crosstalk refers to directional communication in which signals produced or displayed by one cell type alter the other cell type. These signals include soluble mediators, cell-surface interactions, extracellular traps, metabolites, and extracellular vesicles (EVs).

The subsections below organize the evidence according to the inflammatory context examined in each study. Findings obtained in the presence of type 2 cytokine activity or eosinophilic allergic inflammation are discussed under type 2-high mechanisms, whereas those associated with neutrophilic or non-type 2 responses are considered relevant to type 2-low asthma, without establishing exclusive endotype specificity. Because type 2 and non-type 2 responses may coexist in severe asthma, findings from mixed-granulocytic models are described as representing overlapping inflammatory features rather than a distinct endotype; for organizational clarity, pathways examined in mixed-granulocytic settings are discussed in the section relevant to type 2-low asthma when no clearly dominant type 2 phenotype has been established in relevant studies. When the same molecule has been investigated separately in type 2-high and type 2-low settings, the context-specific findings are discussed in the corresponding subsections. Further details are summarized in **Table 1**. The major signaling axes and implicated macrophage populations are illustrated in **Figure 1**.

**Table 1:** Major signaling and evidence summary of AEC–macrophage crosstalk in asthma.

Direction and signaling	Source cells	Recipient cells	Asthma endotype	Disease/model	Downstream effects in human evidence	Downstream effects in experimental evidence	Level and main limitation
AEC→Macrophage TSLP/TSLPR	AECs	CD206^+^ sputum macrophages	Type 2-high	Stable adult asthma cohort	In patients with asthma, a higher proportion of TSLPR+ cells in CD206^+^ sputum macrophages was associated with longer disease duration and impaired lung function (123). (association evidence)	NA	**III. Association or indirect evidence.** Human sputum data link macrophage TSLPR expression to clinical features, but do not demonstrate epithelial TSLP release, direct AEC-to-macrophage signaling, or macrophage TSLPR necessity.
AEC→Macrophage TSLP—AMFR	AECs as the contextual TSLP source; mechanistic experiments used exogenous TSLP	Murine AMs, bone marrow-derived macrophages	Type 2-high	Allergic asthma mouse model; isolated mouse macrophage stimulation	NA	TSLP–AMFR–STAT5–GM-CSF axis upregulated in AMs of asthmatic mice; AMFR deficiency attenuated inflammatory and asthmatic responses via controlling AMs and macrophage genetic loss-of-function (124).	**IIa. *In vivo* necessity.** Amfr loss reduces AM-derived GM-CSF and allergic inflammation; AMFR is required for TSLP-STAT5 signaling in macrophages, but TSLP was exogenous or contextual; AEC-specific TSLP necessity and human validation are absent.
AEC→Macrophage IL-33/ST2	AECs; lung epithelial cells	CD206^+^ sputum macrophages, murine AMs, bone marrow-derived macrophages, human peripheral blood monocyte-derived macrophages	Type 2-high	Human atopic asthma cohort; allergic asthma mouse model; isolated mouse and human macrophage stimulation	Bronchial and lung epithelial IL-33^+^ cells were more abundant in asthma than in control tissue (125). (biomarker evidence); ST2 expression on CD206^+^ sputum macrophages was associated with disease duration and impaired lung function (123). (association evidence)	IL-33/ST2 signaling enhanced IL-13-dependent alternative macrophage activation and increased type 2 immune responses (125). Guo et al. Myeloid ST2 deletion and adoptive-transfer experiments in mice reduced M(IL-33+IL-2) macrophages and eosinophilic inflammation; JAK2–STAT3–IRF1 perturbation supported the downstream pathway (126).	**IIa. *In vivo* necessity.** ST2 deletion and adoptive-transfer experiments support that macrophage ST2 is required for direct responses to IL-33, but the IL-33 source was not AEC-specifically perturbed in the same asthma model, and the gene deletion was not macrophage-exclusive.
AEC→Macrophage IL-25/IL-17RA	AECs as the contextual IL-25 source; mechanistic experiments used exogenous IL-25	CD206^+^IL-17RA^+^ sputum macrophages, macrophages isolated from murine lung tissue, THP-1 cells and THP-1 derived macrophages	Type 2-high/Type 2-low	Human atopic and eosinophilic/non-eosinophilic asthma cohorts; neutrophil-/eosinophilic-dominant allergic asthma mouse model; THP-1 culture	CD206^+^IL-17RA^+^ sputum macrophages were increased with higher sputum IL-25, IL-13, and eosinophils in atopic asthma (127). (association evidence); Lower IL-25 in severe/non-eosinophilic asthma was associated with increased M1 macrophages (140). (association evidence)	Recombinant IL-25 induced ROS production, AMPK activation, PINK1–Parkin-mediated mitophagy in THP-1-derived macrophages, and was associated with M2 macrophage polarization (128). Exogenous IL-25 reduced expression of IL-12 and IL-23, lung macrophage M1 markers, and airway neutrophilia in mice (140).	**IIb. Partial necessity.** Macrophage responses to IL-25 and downstream-pathway inhibition support ROS-AMPK-PINK1/Parkin pathway dependence. Endogenous AEC-derived IL-25 and macrophage IL-17RA were not tested for necessity *in vivo*; THP-1 and THP-1-derived macrophages limit inference.
AEC→Macrophage CCL2/CCR2	AECs (human tissue and BEAS-2B)	CCR2-responsive recruited inflammatory monocytes and monocyte-derived airway/BAL macrophages	Type 2-high	Human mild asthma after allergen challenge; allergic asthma mouse model; cynomolgus monkey asthma model; *in vitro*	Allergen challenge in mild allergic asthma increased BAL CCL2 and recruited smaller CD14highCD11bhighCD163^+^ macrophages; BAL macrophage CCR2 and M2-associated transcripts increased (91). (association evidence)	HDM stimulated BEAS-2B CCL2, mouse model showed epithelial CCL2 with recruited macrophages; broad macrophage depletion reduced inflammation/remodeling (91). CCL2/CCR2 blockade reduced inflammatory-monocyte recruitment and Th2 inflammation in mice (131). Anti-CCR2 antibody reduced macrophage/eosinophil influx, AHR, and lung dysfunction in allergic cynomolgus monkeys (132).	**IIa. *In vivo* necessity.** CCR2 blockade reduces inflammatory monocyte/macrophage recruitment and allergic inflammation across animal models, consistent with epithelial CCL2 induction. AEC-specific CCL2 deletion and macrophage-restricted CCR2 testing are lacking; other CCR2-positive cells may contribute.
AEC→Macrophage OSM/OSMR	AECs	Airway macrophages	Type 2-high	MNP plus OVA induced allergic asthma mouse model; isolated AEC–macrophage co-culture	NA	MNP induced epithelial ER stress and OSM. Exogenous OSM or MNP-primed AECs increased airway macrophage TNF-α through OSM–OSMR signaling. PERK inhibition and macrophage Osmr depletion attenuated allergic airway inflammation (133).	**IIa. *In vivo* necessity.** AEC-derived OSM and macrophage OSMR-dependent responses are supported, and macrophage-lineage Osmr loss attenuates disease *in vivo*. AEC-specific Osm deletion was not performed; human validation is absent.
AEC→Macrophage TIMP-1—CD63	AECs (primary SAECs and A549 cells)	Human peripheral blood monocyte-derived macrophages; murine lung macrophages; eosinophils	Type 2-high	Human asthma cohort; chronic severe allergic asthma mouse model; vitro	Serum TIMP-1 was elevated in severe/type 2 severe asthma and associated with reduced FEV1 and increased EDN/VEGF levels (135). (association evidence)	Stimulated AECs released TIMP-1. Recombinant TIMP-1 induced CD63-dependent M2-like macrophage polarization and VEGF release; anti-TIMP-1 attenuated eosinophilic inflammation, M2-like lung macrophages, VEGF, and remodeling in chronic OVA-challenged mice (135).	**IIb. Partial necessity.** AEC TIMP-1 release and CD63-dependent macrophage responses are supported, and systemic TIMP-1 blockade improves disease outcomes. AEC-specific TIMP-1 and macrophage-specific CD63 necessity were unverified; eosinophils are parallel recipients.
AEC→Macrophage CD200/CD200R	AECs; alveolar type II epithelial cells	Murine CD200R-high resident AMs; other CD200R^+^ immune cells	Type 2-high; influenza- and LPS-associated non-asthma contexts	Allergic asthma rat model; Influenza-associated lung inflammation mouse model; vitro	NA	Epithelial cells released CD200 suppressed AM IL-6/TNF production. CD200/CD200R loss aggravated influenza inflammation; CD200/CD200R activation restored lung inflammatory control (137). Airway recombinant CD200 abolished AHR, reduced lung DC/Th2 accumulation and BAL IL-13, and increased IL-10 in rats (139).	**IIb. Partial necessity.** Epithelial CD200 suppresses CD200R-dependent macrophage cytokines mainly *in vitro* and in influenza/LPS models. Recombinant CD200 reduced asthma features, but endogenous AEC-macrophage signaling and cell-specific necessity were unverified; other CD200R^+^ immune cells may be involved in asthma.
AEC→Macrophage IL-17C/IL-17RE	AECs; serum	IL-17RE^+^ recruited monocytes and lung macrophages; RAW264.7 cells	Type 2-low (type-17 inflammation)	Human moderate/severe asthma cohorts; OVA/CFA steroid-insensitive neutrophilic asthma mouse model; vitro	Bronchial epithelial IL-17C was higher in severe than moderate asthma and inversely associated with lung function; sputum IL-17RE was higher in severe asthma (65). (association evidence)	IL-17C released after rhinovirus stimulation in differentiated AECs (141). In OVA/CFA neutrophilic asthma, IL-17C increased CCL2, recruited IL-17RE^+^ monocytes and supported M1 macrophage polarization; Il17c deficiency reduced these responses and type-17 inflammation. IL-17RE overexpression in RAW264.7 cells altered STAT1/STAT6-associated polarization responses (65).	**IIa. *In vivo* necessity.** Il17c loss reduces IL-17RE^+^ monocyte/macrophage recruitment, M1 polarization, and type 17 inflammation. AEC-specific Il17c deletion and macrophage-specific IL-17RE loss were not performed; source and recipient evidence were shown across systems.
AEC→Macrophage SAA1	AECs (primary SAECs and A549 cells)	Human peripheral blood monocyte-derived macrophages	Type 2-low	Adult human asthma cohort; OVA ± poly(I:C) eosinophilic/neutrophilic/mixed granulocytic asthma mouse models	Serum SAA1 was higher in asthma, especially neutrophilic/severe disease, and correlated with sputum neutrophilia and lower lung function (144). (association evidence)	Poly(I:C) induced SAA1 release from primary SAECs/A549 cells, further enhanced by neutrophils. In separate assays, recombinant SAA1 induced iNOS^+^CD86^+^ macrophages and M1-associated proinflammatory cytokines in human monocyte-derived macrophages (144).	**III. Association or indirect evidence.** Separate assays show AEC SAA1 release and recombinant SAA1-induced macrophage polarization, but direct co-culture, SAA1 loss-of-function, or cell-specific *in vivo* validation was not performed.
AEC→Macrophage S100A9	AECs (primary SAECs and A549 cells)	Human peripheral blood monocyte-derived neutrophils and macrophages	Type 2-high/Type 2-low	Adult neutrophilic asthma cohort; eosinophilic/neutrophilic/mixed asthma mouse models; Alternaria-induced type 2-high mouse model; coculture of A549 cells and macrophages	Serum S100A9 was elevated in neutrophilic asthma, correlated positively with sputum neutrophils and inflammatory markers (145). (association/biomarker evidence).	AECs released S100A9. Recombinant S100A9 induced inflammatory cytokines and iNOS^+^ macrophages, attenuated by anti-S100A9. Co-culture and neutrophilic-asthma models linked S100A9 to epithelial remodeling and neutrophilic inflammation (145). S100A9 deficiency worsened Alternaria-induced type 2 inflammation via impaired Treg suppression, without macrophages reported in this context (146).	**IIb. Partial necessity.** AEC-derived S100A9, macrophage responses to recombinant S100A9, co-culture effects, and *in vitro* anti-S100A9 inhibition support an AEC-macrophage link. AEC-specific S100A9 deletion and macrophage-specific receptor/pathway necessity were unverified. S100A9 shows different effects across asthma types.
AEC→Macrophage soluble E-cadherin	AECs	THP-1 derived macrophages	Type 2-low (severe asthma)	Human mild to moderate asthma/severe asthma cohorts; TDI- and OVA/CFA-induced severe asthma mouse models; vitro	Sputum soluble E-cadherin was increased in severe versus mild-to-moderate asthma/controls and correlated with sputum HMGB1 and glucocorticoid dose (147). (association evidence).	Recombinant sE-cadherin induced ERK-dependent VEGF production in THP-1-derived macrophages. E-cadherin neutralization reduced mixed-granulocytic inflammation, AHR, and smooth-muscle thickening in mice; sE-cadherin-induced airway inflammation was attenuated by ERK inhibition (147).	**IIb. Partial necessity.** *In vivo* sE-cadherin neutralization and ERK inhibition reduce airway inflammation; recombinant sE-cadherin induces macrophage VEGF. The epithelial source and macrophage-specific signaling requirement were not established *in vivo*; THP-1 cells limit evidence.
Macrophage→AEC PGRN	Murine AMs; MH-S cells	Murine AECs; MLE-12 cells	Type 2-high	Allergic asthma mouse model;vitro	NA	HDM induced early PGRN production predominantly in AMs. Myeloid Grn deletion reduced BALF IL-33, TSLP, IL-4, and IL-13, and allergic airway inflammation in mice. Sensitization-phase PGRN rescue restored type 2 airway inflammation. Intranasal recombinant PGRN increased IL-33/TSLP levels in murine BALF, and direct PGRN stimulation increased IL-33/TSLP from MLE-12 epithelial cells *in vitro (*149).	**IIa. *In vivo* necessity.** Grn deletion and sensitization-phase PGRN rescue support an *in vivo* requirement for macrophage-derived PGRN. Experiments also support a directional epithelial response. However, epithelial-specific PGRN signaling was unverified; human evidence is lacking.
Macrophage→AEC CD30L/CD30	Human lung CD68^+^ macrophages; murine F4/80^+^ macrophages; THP-1-derived macrophages; murine bone marrow-derived macrophages	AECs (human and mouse bronchial epithelial cells)	Type 2-high	Human asthma biopsy/serum cohorts; allergic asthma mouse model; coculture of THP-1-derived macrophages and human bronchial epithelial cells	Airway CD30L was increased and colocalized with CD68^+^ macrophages in asthma biopsies. Serum CD30L was elevated in moderate/severe asthma and inversely correlated with FEV1/FVC and FEV1% predicted (153). (association evidence)	In asthmatic mice, macrophage-lineage CD30L gene deletion reduced epithelial EMT, mucus hypersecretion, collagen deposition, and AHR; exogenous CD30L exacerbated these features. In indirect macrophage–AEC coculture, macrophage CD30L silencing attenuated epithelial remodeling and migration; epithelial CD30 silencing or JNK/p38 inhibition reversed the CD30L-induced effects, supporting the CD30L-CD30-JNK/p38 axis (153).	**I. Causal evidence.** Macrophage-lineage CD30L gene deletion and CD30L gain-of-function alter epithelial remodeling *in vivo*; macrophage-conditioned medium and epithelial CD30/JNK/p38 perturbation establish direction and recipient mechanism. Epithelial CD30 necessity remains untested *in vivo*.
Macrophage→AEC 27-HC	Murine AMs	AECs (BEAS-2B, primary AECs); MLE-12 cells	Type 2-high	Allergic asthma mouse model; separate isolated-AM and AEC stimulation	NA	AMs are the predominant 27-HC source; IL-33 induced AM 27-HC production via NF-κB. In AECs, exogenous 27-HC induced ROS-dependent apoptosis and AMPK-dependent impairment of efferocytosis; AMPK inhibition partially restored efferocytosis. Intranasal 27-HC worsened eosinophilic inflammation and AHR; CYP27A1 inhibition reduced lung 27-HC and disease severity *in vivo (*156).	**IIb. Partial necessity.** AMs are the predominant 27-HC source, exogenous 27-HC impairs AEC efferocytosis, and CYP27A1/AMPK pharmacology supports pathway involvement. But AM-specific Cyp27a1/27-HC loss-of-function and epithelial-specific AMPK necessity were not tested; direct transfer/co-culture and human evidence are lacking.
Macrophage→AEC OSM/OSMR	Human lung macrophages; human peripheral blood monocyte-derived macrophages; murine bone marrow-derived macrophages	AECs (BAEC-ALI); murine lung epithelial cells	Type 2-low (severe asthma)	Human severe-asthma cohort; HDM, HDM/LPS, acute LPS and K. pneumoniae mouse models; vitro	Severe-asthma bronchial brushings showed OSM was the top predicted upstream regulator, epithelial OSMR was increased and correlated with IL-33 and mucus-related expression (157). (association evidence)	Exogenous OSM exacerbated HDM-induced inflammation, and Osm deficiency or blockade reduced inflammation and mucin expression in mouse models; an intratracheal transfer experiment supported macrophage-derived OSM involvement *in vivo*. LPS induced macrophage OSM secretion *in vitro*; recombinant OSM increased mucus production and impaired mucociliary clearance in AECs (157).	**IIa. *In vivo* necessity.** Macrophage-derived OSM is supported by macrophage-transfer and OSM-blocking experiments, and exogenous OSM directly alters AEC responses. However, OSMR requirement in AECs was not tested; other OSM-responsive cells may contribute.
Macrophage→AEC METs	Human peripheral blood monocytes and monocyte-derived macrophages	AECs (A549 and 16-HBE cells)	Type 2-low (chronic/severe asthma)	Human severe versus nonsevere asthma cohort; HDM+LPS-induced chronic asthma mouse model; vitro	MoETs and M1ETs were increased in severe asthma greater than nonsevere asthma, and correlated positively with blood neutrophils, MCP-1, and soluble ST2 and negatively with FEV1% (159). (association evidence).	MoETs/M1ETs induced inflammatory mediators and barrier loss in A549 cells; intranasally administered MoETs/M1ETs increased BALF neutrophils and lung inflammation (159). HDM-induced METs from human monocyte-derived macrophages promoted 16-HBE migration and EMT via NF-κB/ERK1/2; Metrnl/IL-41 partly reversed these effects *in vitro*; reduced neutrophils, M1, and Th17 cells in the lungs of asthmatic mice (160).	**IIb. Partial necessity.** MET exposure directly alters AECs *in vitro* and increases airway inflammation *in vivo*; Metrnl/IL-41 reduces MET formation and remodeling. No MET-selective or macrophage-specific trap perturbation establishes endogenous necessity, and Metrnl/IL-41 affects multiple cell types and pathways.

This table summarizes experimentally and clinically reported AEC–macrophage crosstalk pathways in asthma, including signaling direction, cellular sources and recipients, asthma endotypes, disease models, downstream effects, and evidence strength. Evidence is graded for the proposed intercellular axis, rather than for the mediator’s overall involvement in asthma, including Level I causal evidence, Level II necessity evidence, and Level III association or indirect evidence.

I. Causal evidence indicates that both source-to-recipient signaling and functional consequences are demonstrated with cell-specific mechanistic validation. Cell- or lineage-targeted loss-of-function on at least one side links this interaction to concordant recipient and disease outcomes *in vivo*.; IIa. *In vivo* necessity indicates that an endogenous ligand, receptor, or source/recipient component is required *in vivo*, although complete cell-specific validation of both sides of the signaling axis may be incomplete; IIb. Partial necessity indicates that pathway inhibition, blockade, loss-of-function, or *in vitro* mechanistic evidence supports part of the signaling axis is necessary, but endogenous cell-specific necessity remains unproven; III. Association or indirect evidence indicates correlative, biomarker, or indirect evidence without demonstrated functional necessity.

AM, alveolar macrophage; AEC, airway epithelial cell; BAL, bronchoalveolar lavage; HDM, house dust mite; OVA, ovalbumin; SAECs, small airway epithelial cells; BEAS-2B, human bronchial epithelial cell line; A549, human lung adenocarcinoma epithelial cell line; THP-1, human monocytic leukemia cell line; RAW264.7, murine macrophage cell line; MH-S, murine alveolar macrophage cell line; MLE-12, murine lung epithelial-12 cell line; BAEC-ALI, bronchial airway epithelial cells cultured at an air–liquid interface; 16-HBE, human bronchial epithelial cell line; NR8383, rat alveolar macrophage cell line; M1, M1 macrophages; M2, M2 macrophages; Th17, T helper 17 cells.

Bold text indicates the evidence-level classifications assigned to each proposed AEC–macrophage signaling axis.

Bold text indicates the evidence-level classifications assigned to each proposed AEC–macrophage signaling axis.

**Figure 1:**
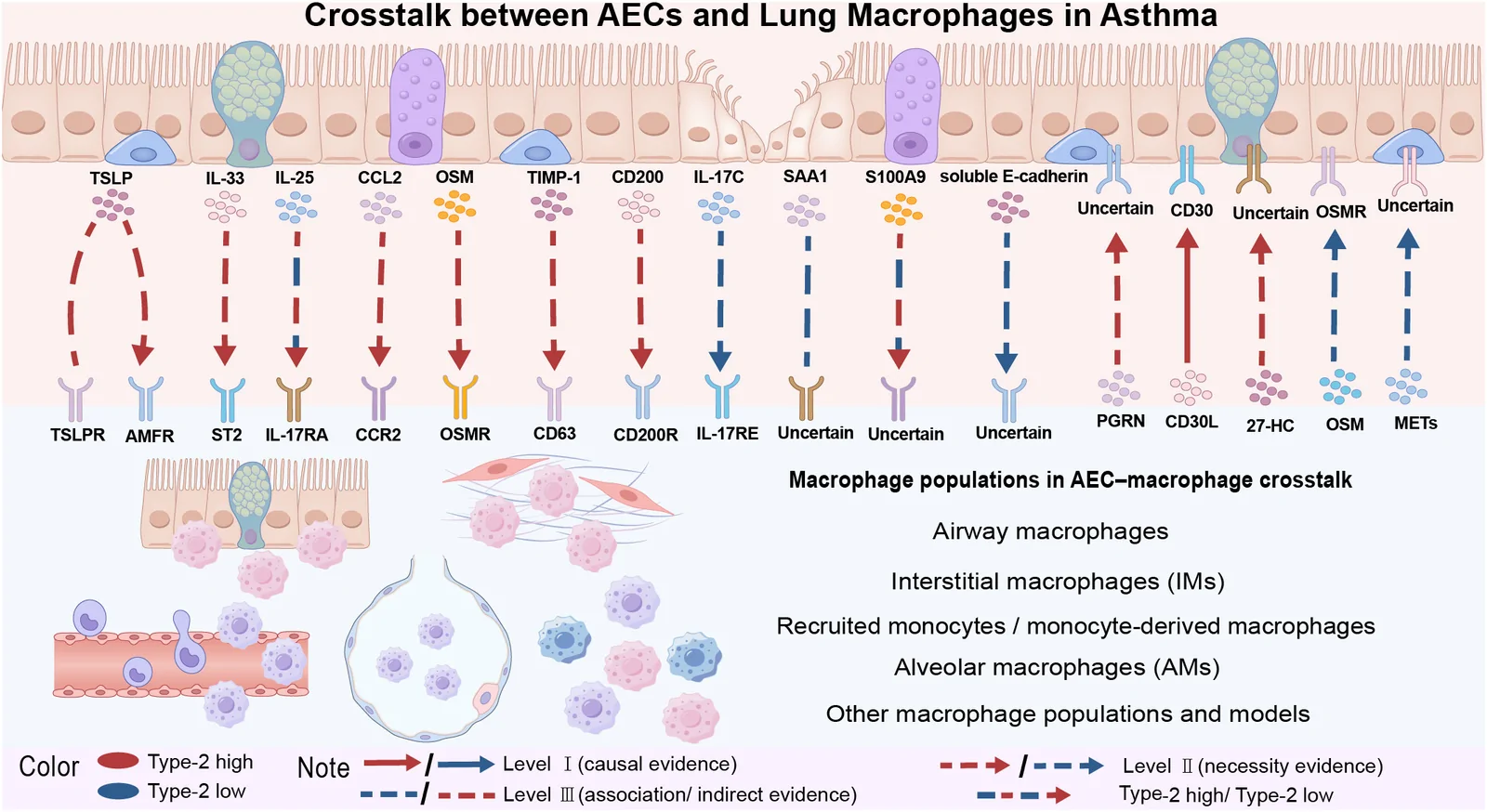
Crosstalk between AECs and lung macrophages in asthma. The figure illustrates major AEC–macrophage signaling axes and implicated macrophage populations. Arrow direction indicates signaling direction; arrow style denotes evidence level (I, causal; II, necessity; III, association/indirect). Red and blue indicate Type 2-high and Type 2-low relevance, respectively; combined colors indicate Type 2-high or Type 2-low relevance. “Uncertain” denotes an unresolved interacting component.

### 5.1 AEC-to-macrophage in asthma

AECs participate in the initiation of asthmatic inflammation. They can also regulate lung macrophage recruitment, activation, and effector functions.

#### 5.1.1 Mechanisms associated with type 2-high inflammation

1. AEC-Derived Alarmin Effects on Macrophages: AEC-derived alarmins can influence macrophages both indirectly through type 2 immune networks and directly through macrophage receptors (122). In patients with asthma, TSLPR and ST2 expression on CD206^+^ sputum macrophages were associated with disease duration and impaired lung function, supporting their capacity to directly respond to epithelial alarmins (123). In mice, after TSLP stimulation, autocrine motility factor receptor (AMFR) was mainly upregulated in AMs and promoted STAT5 activation and granulocyte-macrophage colony-stimulating factor (GM-CSF) production, whereas AMFR deficiency attenuated these TSLP-driven responses and reduced Th2 and eosinophilic inflammation (124).
  IL-33/ST2 signaling promotes IL-13-dependent alternative activation and CCL17/CCL24 production in murine alveolar and bone marrow-derived macrophages, promoting airway inflammation and eosinophilia (125). Guo et al. identified an IL-9-producing macrophage state, termed M(IL-33+IL-2), in murine lungs and human monocyte-derived macrophages, which enhanced eosinophilic inflammation and mucus secretion experimentally (126).
  IL-25 provides a context-dependent example. Gryz et al. found that increased CD206^+^IL-17RA^+^ sputum macrophages were accompanied by higher airway IL-25 levels and type 2 inflammatory features in atopic asthma (127). *In vitro*, recombinant IL-25 increased ROS production and mitochondrial respiratory chain complex activity, subsequently activated AMPK, and induced mitophagy to promote M2 polarization (128). IL-25 effects in neutrophilic inflammation are discussed in Section 5.1.2.
2. CCL2/CCR2: CCL2, also known as MCP-1, recruits monocytes and macrophages primarily through C-C chemokine receptor 2 (CCR2) (129, 130). In patients with mild allergic asthma, allergen challenge increased BAL CCL2 and recruited smaller CD14^high^CD11b^high^CD163^+^ macrophages, accompanied by increased CCR2 expression on AMs and M2-associated proteins (91). HDM directly stimulated CCL2 production by bronchial epithelial cells *in vitro (*91). In an allergic mouse model, epithelial CCL2 promoted the recruitment of circulating monocytes and the expansion of monocyte-derived AMs, thereby exacerbating type 2 inflammation and airway remodeling (91). Across several animal models of asthma, blockade of CCL2/CCR2 signaling reduced recruitment of inflammatory monocytes/macrophages and eosinophils, suppressed Th2 responses, and attenuated allergic airway inflammation and AHR (131, 132).
3. OSM/OSMR: In an OVA-induced mouse model, the environmental pollutant 3-methyl-4-nitrophenol (MNP) increased epithelial endoplasmic reticulum (ER) stress and oncostatin M (OSM) production (133). OSM is a member of the IL-6 cytokine family, whose receptors share the glycoprotein 130 subunit (134). Both exogenous OSM and MNP-stimulated AECs increased TNF-α production by mouse lung-derived macrophages through OSM–OSMR signaling. PERK (a key mediator of ER stress) inhibition or macrophage OSMR depletion attenuated allergic airway inflammation (133).
4. TIMP-1—CD63: Tissue inhibitor of metalloproteinases 1 (TIMP-1), majorly produced by AECs in response to diverse stimuli, is a secreted glycoprotein that inhibits airway metalloproteinases, limits ECM degradation, and promotes matrix accumulation. Serum TIMP-1 is increased in type 2-high asthma and is associated with disease severity (135). In separate functional experiments, stimulated AECs released TIMP-1, and recombinant TIMP-1 induced M2-like phenotypes and VEGF release in human monocyte-derived macrophages, which were attenuated by CD63 blockade. TIMP-1 neutralization inhibited M2-like polarization and eosinophilic inflammation in chronic allergic mice (135).
5. CD200/CD200R: CD200, formerly known as OX-2, is an immunoglobulin superfamily transmembrane glycoprotein. Airway epithelial surface CD200 can restrain AM activation through CD200R, whereas evidence linking this interaction to macrophage quiescence and inflammatory control derives mainly from homeostatic and influenza-associated experiments rather than asthma-specific source–recipient perturbations (136, 137). CD200 deficiency promotes lung macrophage accumulation, increases MHC II and CD80 expression on AMs, and enhances macrophage IL-6 and TNF signaling in response to LPS stimulation (137). During inflammation, epithelial CD200 remains relatively stable or transiently decreases, while macrophage CD200R increases gradually and peaks during inflammatory resolution, supporting a role for CD200–CD200R signaling in limiting excessive macrophage activation and restoring immune homeostasis (137, 138). In an acute allergic asthma rat model, recombinant CD200 reduced BALF IL-13, lung DC/Th2-cell accumulation, and AHR, without significantly reducing eosinophil recruitment (139); these results can serve as inhibitory evidence of AEC-to-macrophage interactions in asthma, but effects on other CD200R+ cells remain possible.

#### 5.1.2 Mechanisms relevant to type 2-low asthma

1. IL-25: Lower IL-25 in severe/non-eosinophilic asthma was associated with increased M1 macrophages. In neutrophilia-dominant mice, exogenous IL-25 ameliorated airway neutrophilia via suppressing M1 polarization and IL-12/IL-23 expression (140). Together with Section 5.1.1, these findings support context-dependent macrophage regulation, rather than a uniformly pro-inflammatory action.
2. IL-17C/IL-17RE: AECs can release basolateral IL-17C after pathological stimulation like rhinovirus (141). IL-17C is an epithelial-associated member of the IL-17 cytokine family and mainly binds to a heterodimeric receptor complex composed of IL-17RE and IL-17RA, with IL-17RE recognizing IL-17C specifically (142, 143). In a human cohort, bronchial epithelial IL-17C expression is increased in severe compared with moderate asthma, and circulating IL-17C is correlated with neutrophils and with impaired lung function (65). In an OVA/CFA-induced neutrophilic asthma mouse model, IL-17C increased CCL2 expression, promoted the migration of IL-17RE^+^ monocytes into the lung, and supported M1-like macrophage differentiation. IL-17RE^+^ macrophages were the principal responders to IL-17C and exacerbated neutrophil- and type 17-dominant inflammation, whereas Il17c deficiency reduced these pro-inflammatory responses and limited M1-like macrophage expansion (65).
3. SAA1: Serum amyloid A1 (SAA1) expression levels are higher in patients with neutrophilic asthma than in those with non-neutrophilic asthma. It is associated with neutrophilic/severe asthma, sputum neutrophilia, and lower lung function. SAA1 is a multifunctional protein that functions as an acute-phase protein, an apolipoprotein, and an innate immune mediator. *In vitro*, polyinosinic-polycytidylic acid [poly(I:C)] can stimulate AECs to release SAA1, and neutrophils enhance this response. Recombinant SAA1 can independently induce inflammatory cytokines and promote macrophage M1 polarization (iNOS^+^CD86^+^) in human monocyte-derived macrophages, exacerbating neutrophilic inflammation (144).
4. S100A9: Quoc et al. identified S100 calcium-binding protein A9 (S100A9) as a candidate serum biomarker and therapeutic target in neutrophilic asthma. *In vitro*, neutrophils from patients with asthma stimulated epithelial S100A9 release; recombinant S100A9 promoted neutrophil activation, NET formation, and M1-like macrophage polarization, while S100A9 blockade attenuated these responses in asthma mouse models (145). S100A9 appears to be asthma endotype-dependent. The opposing findings in Alternaria-induced type 2 allergic airway inflammation indicated that its deficiency aggravated Th2 inflammation and AHR, suggesting S100A9 can be a protective factor in type-2 high inflammation, but without macrophages reported in this study (146).
5. Soluble E-cadherin: E-cadherin, a guardian of the mucosa, can be cleaved from the cell surface and released in soluble form. Soluble E-cadherin is increased in sputum from patients with severe asthma (147). Recombinant soluble E-cadherin activated the ERK pathway-dependent VEGF and IL-6 responses in THP-1-derived macrophages, while neutralization of soluble E-cadherin attenuated airway inflammation and AHR in an experimental severe asthma model (147). Both neutrophilic and eosinophilic responses were involved in severe asthma, whereas specific inflammatory types remained unrevealed, supporting relevance to overlapping inflammatory features.

### 5.2 Macrophage-to-AEC in asthma

Lung macrophages not only respond to AEC-derived signals but can also directly alter AEC-derived alarmin production, mucus secretion, clearance functions, and remodeling-related responses.

#### 5.2.1 Mechanisms associated with type 2-high inflammation

1. PGRN: Progranulin (PGRN) is a secreted cysteine-rich glycoprotein and growth-regulatory factor, involved in inflammatory regulation and tissue repair (148). Following HDM exposure, airway PGRN increased earlier than other type 2 cytokines and was produced predominantly by AMs. Myeloid Grn deletion reduced allergic airway inflammation in mice and decreased BALF IL-33, TSLP, IL-4, and IL-13, whereas PGRN supplementation during sensitization restored inflammatory responses (149). Recombinant PGRN increased IL-33/TSLP in BALF, and direct stimulation of MLE-12 epithelial cells increased these alarmins (149). PGRN also stimulated NKT-cell IL-4/IL-13 production, and NKT deficiency abolished the PGRN-induced Th2 cytokine response (149). These findings indicate that macrophage-derived PGRN can induce epithelial alarmins during the early phase of allergic sensitization and initiate type 2 inflammation.
2. CD30L/CD30: CD30 ligand (CD30L) is a member of the TNF ligand superfamily and signals through CD30 (150). In allergic airway inflammation, CD30L cooperates with other costimulatory ligands to sustain inflammatory responses (151, 152) and contributes to airway remodeling in asthma (153). CD30L expression is increased in airway biopsies from patients with asthma and in OVA/HDM-challenged mice, where it colocalizes with CD68^+^ or F4/80^+^ macrophages, respectively (153). In asthmatic mice, deletion of the macrophage-lineage CD30L gene reduced epithelial EMT, mucus hypersecretion, collagen deposition, and AHR, whereas exogenous CD30L exacerbated these features. In indirect macrophage–AEC coculture, mechanistically, macrophage CD30L acts on epithelial CD30 to promote ECM deposition and EMT through JNK/p38 MAPK signaling; silencing macrophage CD30L, knocking down epithelial CD30, or inhibiting JNK/p38 attenuates epithelial remodeling and migration (153).
3. 27-HC: 27-Hydroxycholesterol (27-HC), an oxidized cholesterol metabolite and member of the oxysterol family, is primarily generated from cholesterol by sterol 27-hydroxylase (CYP27A1) (154). Aeroallergen exposure increases the CYP27A1–27-HC axis and is associated with allergic inflammation and epithelial tight-junction disruption (154, 155). In an OVA-challenged allergic asthma mouse model, AMs were identified as a major source of 27-HC (156). Mechanistically, airway IL-33 induced macrophage 27-HC production via NF-κB signaling, while exogenous 27-HC impaired epithelial efferocytosis through AMPK activation and worsened eosinophilic inflammation and AHR (156). These findings suggest that a macrophage-derived metabolite can aggravate type 2-related inflammation by weakening epithelial homeostatic clearance in asthma (156).

#### 5.2.2 Mechanisms relevant to type 2-low asthma

1. OSM/OSMR: As discussed above, AEC-derived OSM can signal to macrophages; macrophage-derived OSM can also mediate epithelial responses during bacterial challenge (133, 157). However, the reported OSM signaling directions were established in separate studies and do not demonstrate a closed reciprocal loop. In severe asthma, airway biopsies show an OSM-associated transcriptional profile associated with inflammation and mucus production, and single-cell analyses identify lung macrophages as an important pulmonary source of OSM (157). Separately, LPS stimulated OSM secretion by human monocyte-derived and murine bone marrow-derived macrophages. Recombinant OSM induced mucus-associated responses in primary human AECs, including goblet-cell differentiation and extracellular mucus accumulation, thereby exacerbating bacterial-associated severe asthma pathology (134, 157). In mice, Osm loss or OSM blockade attenuated inflammation and mucus responses across allergen- and bacterial-trigger models; transfer of LPS-stimulated wild-type versus Osm-deficient macrophages supported the macrophage source (157).
2. METs: Macrophage extracellular traps (METs) are extracellular web-like immune structures released by activated macrophages that contain double-stranded DNA, histones, elastase, matrix metalloproteinases, myeloperoxidase, and other components. Although they can contribute to host defense, excessive MET formation may promote tissue injury under pathological conditions (158). Patients with severe asthma show increased monocyte extracellular traps (MoETs) and M1 macrophage-derived extracellular traps (M1ETs), which correlate positively with blood neutrophils and serum MCP-1/soluble ST2 and negatively with FEV1% (159). Experimental results further show that MoETs and M1ETs promote AEC migration and pro-inflammatory cytokine production, supporting an association with asthma severity (159). Feng et al. found that METs promoted migration and EMT-associated changes in 16-HBE cells through epithelial NF-κB and ERK1/2 signaling, while meteorin-like protein/IL-41 partially inhibited these effects and attenuated airway inflammation, AHR, and remodeling in the HDM+LPS-induced chronic asthma mouse model (160).

### 5.3 EV-mediated crosstalk between AECs and lung macrophages in asthma

The term “EVs” refers to lipid bilayer-enclosed particles released by cells that cannot replicate independently. “Exosomes” refers to a biogenesis-related term indicating origin from the endosomal system; it represents a subtype of EVs, generally less than 200 nm in diameter (161). EVs can carry molecular cargo, including proteins, lipids, nucleic acids, and metabolites, and may influence recipient cells through surface ligand–receptor interactions, internalization, or membrane fusion followed by cargo delivery. The endosomal origin of isolated vesicles is often difficult to establish; the general term “EV” is used below, including for preparations described as “exosomes” in the original studies. The evidence for EV-mediated AEC–macrophage crosstalk is summarized in **Table 2**.

**Table 2:** EV-mediated AEC–macrophage crosstalk in asthma.

EV source	Cargo	Target cell	Asthma endotype	Reported effects	Level and main limitation
AEC: IL-13-stimulated BEAS-2B cells; bronchial epithelium in OVA-allergic mice	Not specified	**Macrophage:** THP-1 monocytes; MH-S cells; bronchial and lung monocytes/macrophages	Type 2-high	IL-13 increased epithelial EV release. EV marker proteins mostly localize to bronchial cells and macrophages. Epithelial EVs enhanced monocyte/macrophage proliferation and chemotaxis. GW4869 reduced EV release and alleviated asthmatic features (163).	**IIb. Partial necessity.** GW4869 is not epithelial-specific; the cargo was unresolved, and recipient necessity *in vivo* was unverified.
AEC: OVA-stimulated BEAS-2B cells; primary mouse tracheal AECs	plexin B2 (PLXNB2)	**Macrophage:** RAW264.7; BAL CD100^+^F4/80^+^ macrophages; lung CD100^+^CD45^+^F4/80^+^ macrophages	Type 2-high/Type 2-low	OVA-induced AEC-PLXNB2- enriched EVs, taken up by macrophages, promoted MMP14-dependent CD100 cleavage plus Ccl2/Ccl5/Cxcl2/Il6 expression, worsening AHR and inflammation. CD100-knockdown macrophage transfer reduced inflammatory recruitment *in vivo*; macrophage Mmp14 knockdown blocked CD100 shedding and chemokine induction *in vitro (*164).	**IIa. *In vivo* necessity.** EV epithelial source and macrophage recipient are supported *in vivo*, but experiments used exogenous EV delivery; endogenous AEC-specific PLXNB2 necessity are lacking.
AEC: Poly(I:C)/rhinovirus stimulate-BEAS-2B cells; primary human bronchial epithelial cells	Tenascin-C (TN-C); functional inflammatory cargo not identified	**AEC:** BEAS-2B cells; **Macrophage:** human monocyte-derived macrophages tested only with recombinant TN-C	Virus-associated asthma exacerbation (endotype not defined)	Poly(I:C) or rhinovirus induced pro-inflammatory epithelial small EVs, including TN-C-associated sEV release. Stimulated-cell sEVs induced CXCL8, IL-6, and CCL5 in BEAS-2B cells, but TN-C knockdown did not reduce CXCL8/CCL5 responses. Recombinant TN-C separately induced macrophage CXCL8 release (165).	**III. Association or indirect evidence.** EV function was shown in epithelial-epithelial *in vitro*; macrophage results used soluble recombinant TN-C but not EVs. TN-C knockdown indicated other functional inflammatory cargo.
AEC: PM2.5-exposed BEAS-2B cells	miR-155-5p	**Macrophage:** PMA-differentiated THP-1 derived CD11b^+^ macrophages	Not asthma model	PM2.5 increased BEAS-2B EV miR-155-5p. Co-culture with macrophages through miR-155-5p/SOCS1/NF-κB pathway promoted the inflammatory response and increased macrophage IL-1β, IL-6, and TNF-α. GW4869 attenuated these responses in co-culture (166).	**IIb. Partial necessity.** Coculture *in vitro* supports AECs and macrophages’ necessity; GW4869 supports EV involvement, but it’s nonspecific; miR-155-5p dependence and asthma relevance are unverified.
Macrophage: Murine AMs	SOCS3: (microparticles /EVs)	**AEC:** BEAS-2B cells (168); Rat alveolar epithelial cells (167)	Type 2-high (168); Not asthma model (167)	BALF and AM-secreted SOCS3 were reduced in asthma/allergen models; AM EVs suppressed epithelial STAT3/STAT6 signaling; SOCS3 liposomes attenuated OVA-induced inflammation (168). AM-derived SOCS3-containing microparticles were taken up by alveolar epithelial cells and inhibited STAT signaling. SOCS3 knockdown or microparticle depletion abolished this inhibition *in vitro*. Intrapulmonary AM microparticles reduced STAT activation *in vivo (*167).	**IIb. Partial necessity.** Donor SOCS3 knockdown and microparticle depletion support necessity *in vitro* using alveolar epithelial recipients; *in vivo* studies used exogenous microparticles or synthetic liposomes. Endogenous AM–AEC crosstalk necessity in asthma remains unverified.
Macrophage: IL-4-polarized MH-S cells (M2: F4/80^+^CD206^+^)	Not specified	**AEC:** HDM-stimulated MLE-12 cells; lung epithelial in mouse	Type 2-high	MLE-12 cells internalized M2-derived EVs and reduced HDM-induced proliferation, migration, and apoptosis. Intranasal EV administration reduced epithelial injury, collagen deposition, eosinophilic inflammation, and IL-4/IL-5/IL-13 levels in HDM-challenged mice (169).	**III. Association or indirect evidence.** M2-derived EV uptake and protective effects support functional sufficiency, but no loss-of-function establishes endogenous macrophage–epithelial EV necessity. Functional cargo and epithelial cell-specific mediation remain unverified.
Macrophage: IL-4-polarized mouse macrophages (M2)	circ-Eif3c	**AEC:** LPS-stimulated alveolar epithelial cells	Type 2-high	M2-EV circ-Eif3c attenuated OVA-induced inflammation, apoptosis, and fibrosis; donor circ-Eif3c knockdown diminished, whereas overexpression enhanced, protection. Separately, epithelial circ-Eif3c reduced miR-15a-5p and increased GSS/SOCS6, while miR-15a-5p overexpression or GSS/SOCS6 knockdown reversed protection against LPS-induced apoptosis and ROS accumulation (170).	**IIb. Partial necessity.** Cargo experiment supports administered-EV activity. Direct EV-mediated cargo transfer and endogenous macrophage–AEC necessity remain unproven.
Macrophage: MH-S cells	miR-30a-5p	**AEC:** Mouse bronchial epithelial cells (IL-13/IL-4-exposed)	Type 2-high	Bronchial epithelial cells internalized MH-S EVs; miR-30a-5p-enriched EVs increased recipient miR-30a-5p and suppressed EMT by directly targeting Runx1 and inhibiting RUNX1/TGF-β1 signaling. GW4869 weakened co-culture protection; epithelial RUNX1 or TGF-β1 overexpression reversed protection (171).	**IIb. Partial necessity.** EV blockade and recipient-pathway perturbations support macrophage-AEC crosstalk *in vitro*, but GW4869 is not epithelial-specific. OVA-mouse findings were associative; AM-EV/miR-30a-5p and AEC recipient necessity were unverified *in vivo*.
Macrophage: LPS-stimulated NR8383 rat AMs	miR-21-5p	**AEC:** Rat tracheal epithelial cells	Type 2-high	Uptake of LPS-stimulated macrophage EVs delivered miR-21-5p to rat tracheal epithelial cells, driving EMT through the miR-21-5p–Smad7–TGF-β1/Smad axis. GW4869, miR-21-5p inhibition, or epithelial TGF-β1 knockdown attenuated this response; Smad7 knockdown weakened miR-21-5p-inhibitor protection (172).	**IIb. Partial necessity.** EV blockade, miRNA inhibition, and recipient-pathway perturbations were confined *in vitro*; OVA-rat observations did not establish endogenous macrophage–epithelial EV necessity *in vivo*.

This table summarizes EV-mediated AEC–macrophage crosstalk in asthma, including EV source, cargo, target cell, endotype, reported effects, and major limitations. Evidence levels were assigned according to the criteria defined in **Table 1**. EV is used as the general term, including vesicles originally reported as “exosomes” unless their endosomal origin was demonstrated.

Bold text identifies cell-role labels and evidence-level classifications.

#### 5.3.1 AEC-derived EVs

In homeostatic mouse lungs, approximately 80% of detectable BALF EVs originated from the airway epithelium, whereas allergic airway inflammation changes both their cellular origins and microRNA cargos (162). In allergic asthma models, IL-13 increased epithelial EV release, promoting the proliferation and chemotaxis of undifferentiated monocytes/macrophages *in vitro*; inhibition of EV production with GW4869 reduced the number of proliferating lung monocytes and attenuated airway inflammation in allergic mice (163). OVA challenge similarly increased AEC-derived EVs enriched in plexin B2 (PLXNB2; a natural CD100 ligand), which interacted with CD100^+^F4/80^+^ macrophages, promoted MMP14-dependent CD100 cleavage, and increased CCL2, CCL5, CXCL2, and IL-6 expression, thereby aggravating asthmatic airway inflammation. Macrophage-specific Mmp14 knockdown blocked CD100 shedding and chemokine induction *in vitro*, while CD100-knockdown macrophage transfer reduced inflammatory recruitment *in vivo*, suggesting potential therapeutic targets for EV-mediated asthma exacerbations (164).

AEC-derived EVs may also contribute to infection- and pollutant-associated exacerbations. Poly(I:C) stimulation or rhinovirus infection induced tenascin-C (TN-C) release from bronchial epithelial cells. Poly(I:C)-stimulated BEAS-2B cells also released TN-C-associated small EVs that induced CXCL8, IL-6, and CCL5 release from recipient BEAS-2B cells. Approximately 75% depletion of EV-associated TN-C did not reduce CXCL8 or CCL5 responses, suggesting contributions from other EV components. Separately, the recombinant fibrinogen globe-like domain of TN-C (FBG-C) induced CXCL8 release from human monocyte-derived macrophages (165).

*In vitro*, PM_2_._5_ exposure increased epithelial EVs enriched in miR-155-5p and promoted macrophage inflammatory responses, accompanied by reduced suppressor of cytokine signaling (SOCS) 1, enhanced NF-κB activation, and increased IL-1β, IL-6, and TNF-α production. GW4869 attenuated these effects, but the study did not establish miR-155-5p-specific necessity or validate this pathway in animals or human airway samples (166). Collectively, AEC-derived EVs can regulate macrophage functions through context-dependent cargo, and thereby influence asthma pathobiology.

#### 5.3.2 Macrophage-derived EVs

Bourdonnay et al. showed that AMs transferred SOCS1 and SOCS3 to epithelial cells in EVs and microparticles, respectively; SOCS1 inhibited IFN-γ-induced STAT1 activation in alveolar epithelial cells *in vitro*; SOCS3, a negative regulator of JAK–STAT signaling, suppressed cytokine-induced STAT activation (167). Extending this regulatory mechanism to allergic inflammation, Draijer et al. found lower BALF SOCS3 levels in patients with asthma and reduced AM SOCS3 secretion in allergen-challenged mice (168). SOCS3-containing AM EVs suppressed epithelial STAT3/STAT6 activation *in vitro*, and synthetic liposomes encapsulating SOCS3 restored these epithelial inhibitory effects and attenuated allergic airway inflammation in OVA-challenged mice, suggesting an attenuating function of the SOCS3 regulatory mechanism (168). EVs from IL-4-polarized M2 macrophages reduced abnormal proliferation and apoptosis in HDM-exposed epithelial cells *in vitro* and attenuated airway inflammation and epithelial thickening in HDM-challenged mice (169). A separate study reported that circ-Eif3c-containing EVs from M2 macrophages reduced inflammatory cytokine secretion, lung injury, and airway remodeling in OVA-challenged mice, with cell experiments implicating miR-15a-5p regulation and increased GSS/SOCS6 expression (170).

EVs from miR-30a-5p-overexpressing AMs attenuated IL-4/IL-13-induced EMT-associated changes in mouse AECs through a RUNX1-related mechanism *in vitro (*171). Conversely, macrophage-derived EVs can also carry pathogenic cargo. Mechanistic experiments using EVs from the rat macrophage line NR8383 linked miR-21-5p to Smad7 repression and TGF-β/Smad-associated EMT in rat tracheal epithelial cells, while inhibition of EV release or of miR-21-5p attenuated this response, alongside observations in an OVA-induced asthma rat model (172). Thus, macrophage-derived EVs can exert either protective or pathogenic effects on AECs, depending on macrophage activation state and EV cargo composition.

Overall, direct functional evidence for AEC-macrophage EV communication is derived predominantly from *in vitro* and animal models, while human samples currently provide only associations with related mediators or cellular phenotypes, and direct validation across human asthma endotypes is still lacking.

## 6 Multicellular networks extending AEC–macrophage crosstalk in asthma

AEC-macrophage crosstalk is within broader endotype-associated multicellular networks rather than as an isolated binary interaction. Type 2-high asthma predominantly involves DCs, Th2 cells, NKT cells, ILC2s, and eosinophils. Type 2-low asthma more commonly involves DCs, Th17 cells, ILC1s, ILC3s, and neutrophils. These programs can overlap in severe or mixed-endotype asthma (173). AEC–macrophage interactions within type 2-high and type 2-low inflammatory networks and their links to airway remodeling are illustrated in **Figure 2**.

**Figure 2:**
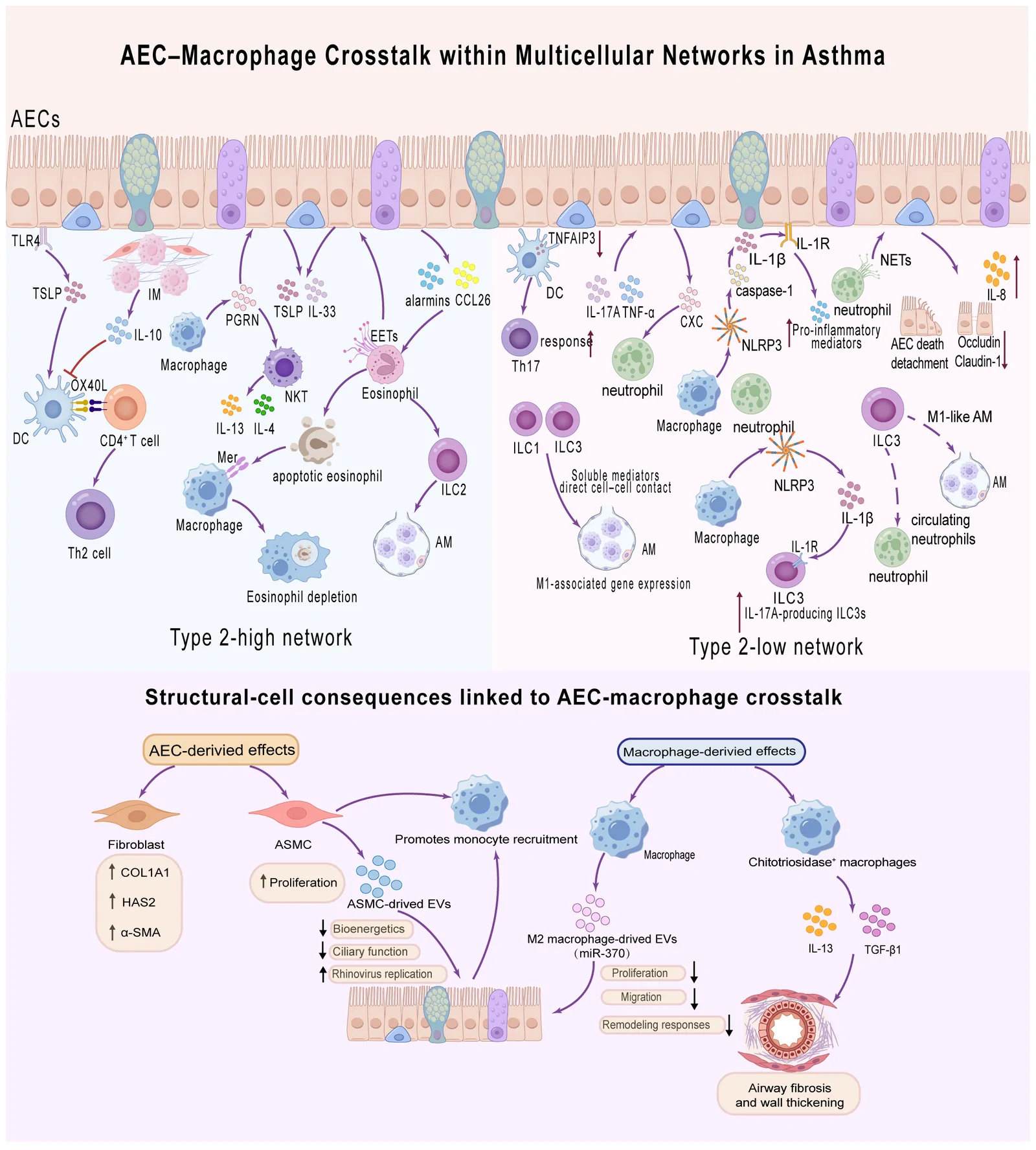
AEC–macrophage crosstalk within multicellular networks in asthma. The figure illustrates AEC–macrophage interactions within type 2-high and type 2-low inflammatory networks and their links to structural-cell responses and airway remodeling. AEC, airway epithelial cell; AM, alveolar macrophage; IM, interstitial macrophage; DC, dendritic cell; ILC, innate lymphoid cell; ASMC, airway smooth muscle cell; EV, extracellular vesicle; EET, eosinophil extracellular trap; NET, neutrophil extracellular trap; NKT, natural killer T cells.

### 6.1 Type 2-high immune networks

After allergen exposure, epithelial TLR4 signaling promotes innate cytokine release, DC activation, and Th2 sensitization (29). TSLP-activated DCs promote Th2 differentiation through OX40 ligand (41), whereas IL-10-producing IMs can restrain DC maturation and migration, thereby limiting Th2 responses and allergic airway inflammation (174). Macrophage-derived PGRN provides another link within this network by inducing epithelial IL-33 and TSLP and promoting IL-4 and IL-13 production by NKT cells (149). Eosinophils further reinforce epithelial–immune feedback. AEC-derived alarmins and CCL26 promote eosinophilic inflammation (175, 176), while eosinophil extracellular traps (EETs), which are positively associated with asthma severity (177), stimulate epithelial IL-33 and TSLP and activate ILC2s in asthma (178). ILC2 abundance positively correlates with M2-like macrophages, and ILC2–AM coculture induces M2-associated gene expression (179). Conversely, macrophage-mediated efferocytosis of apoptotic eosinophils via Mer signaling facilitates the resolution of allergic airway inflammation, whereas Mer inhibition or deficiency delays eosinophil clearance and increases airway responsiveness (180).

### 6.2 Type 2-low immune networks

In type 2-low asthma, DC activation can influence the balance between Th2 and Th17 responses. Reduced TNF-α-induced protein 3 (TNFAIP3) expression in lung DCs promotes HDM-induced Th17/neutrophilic inflammation while suppressing Th2/eosinophilic responses (181). IL-17A and TNF-α subsequently act on AECs to synergistically induce CXCL8 production, thereby promoting neutrophil recruitment (182). In neutrophilic asthma, macrophages and neutrophils express NLRP3 and caspase-1, and sputum IL-1β levels correlate with IL-8 (112). NLRP3 activates caspase-1 to generate mature IL-1β, which signals through epithelial IL-1R to promote pro-inflammatory mediator production (183–185). NETs can aggravate epithelial injury and neutrophilic inflammation by directly inducing AEC death and detachment, disrupting occludin and claudin-1, and increasing epithelial IL-8. NETs are also associated with caspase-1 activation, IL-1β production, mucus hypersecretion, and poorer asthma control (186, 187).

ILC1s and ILC3s are positively correlated with M1-like macrophages, and coculture with AMs induces M1-associated gene expression via soluble mediators and direct cell–cell contact (179). In an obesity-associated asthma mouse model, M1-like lung macrophages increased markedly, and macrophage-derived NLRP3-dependent IL-1β expanded IL-17A-producing ILC3s, whereas IL-1R blockade suppressed ILC3 expansion and AHR (188). Ham et al. found that ILC3s also increased in smoking-associated asthma and correlated with M1-like AMs and circulating neutrophils; *in vitro*, they found that cigarette smoke extract induced ILC3-related responses, disrupted the AEC barrier, and increased epithelial IL-1β production, thereby exacerbating airway inflammation (189).

### 6.3 Structural-cell feedback in airway remodeling

AEC–macrophage crosstalk also extends to fibroblasts and ASMCs, linking persistent inflammation to airway remodeling. In coculture, AECs from patients with asthma increased fibroblast expression of COL1A1, HAS2, and α-smooth muscle actin (α-SMA), with greater responses associated with lung dysfunction or asthma exacerbation (190). HDM-stimulated epithelium also promoted the proliferation of ASMCs from patients with severe asthma (191), whereas ASMC-derived EVs impaired AEC bioenergetics and ciliary function and enhanced rhinovirus replication (192). Rhinovirus-infected epithelium, together with asthmatic ASMCs, further promoted CCL5-dependent monocyte recruitment, linking virus-induced epithelial responses and ASMC activation to the recruitment of macrophage precursors (193). Chitotriosidase-expressing macrophages accumulated and promoted airway fibrosis in chronically HDM-challenged mice, while chitotriosidase inhibition reduced IL-13 and TGF-β1 levels, subepithelial fibrosis, and airway wall thickening (194). M2 macrophage-derived EVs containing miR-370 can suppress ASMC proliferation, migration, and remodeling-associated responses in experimental asthma (195).

## 7 Therapeutic implications

Therapeutic strategies targeting AECs, lung macrophages, and their crosstalk are summarized in **Figure 3** and **Table 3**.

**Figure 3:**
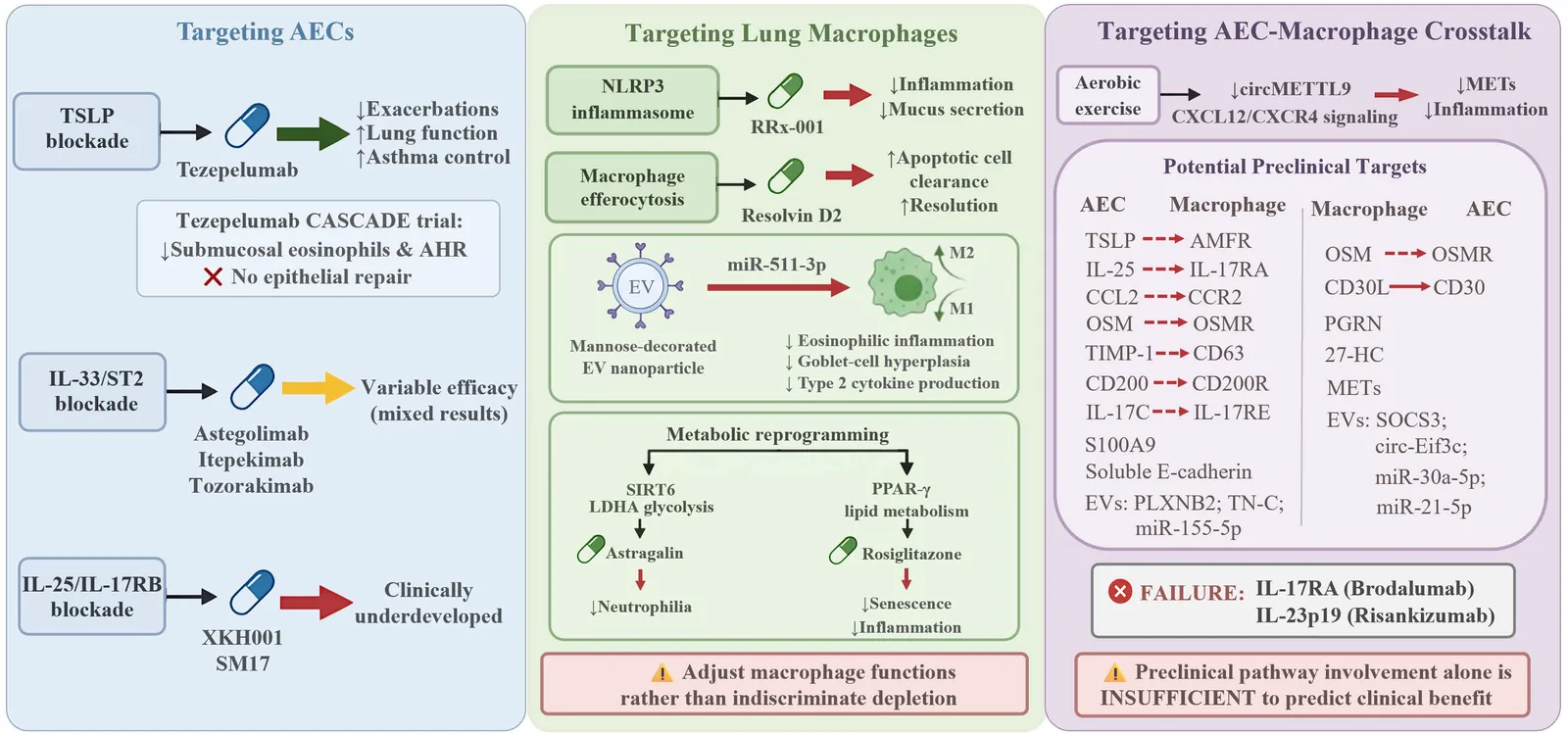
Therapeutic strategies targeting AECs/macrophages/crosstalk in asthma The figure summarizes current and potential interventions targeting AECs, lung macrophages, and their crosstalk, with key mechanisms and therapeutic effects. Clinical evidence is distinguished from preclinical targets, highlighting the translational gap between pathway involvement and clinical benefit. Green, yellow, and red arrows indicate established clinical efficacy, investigational efficacy, and predominantly preclinical evidence, respectively.

**Table 3:** Therapeutic strategies targeting AEC/macrophage/crosstalk in asthma.

Category	Target	Intervention	Mechanism or therapeutic role	Current clinical or experimental evidence
AEC-related	TSLP	Tezepelumab	Neutralizes TSLP and limits downstream airway inflammation	Phase III efficacy and clinical approval; reduces exacerbations across asthma populations
AEC-related	ST2	Astegolimab	Blocks IL-33–ST2 signaling	Phase IIb: reduced exacerbations in severe asthma, including eosinophil-low patients
AEC-related	IL-33	Itepekimab	Neutralizes IL-33 and limits downstream inflammatory signaling	Phase II: fewer loss-of-control events and improved lung function versus placebo in moderate-to-severe asthma
AEC-related	IL-33	Tozorakimab	Blocks reduced IL-33–ST2 signaling and prevents oxidized IL-33–RAGE/EGFR signaling	Phase IIa: primary FEV_1_ endpoint unmet; exploratory benefit in patients with prior exacerbation
AEC-related	IL-25	XKH001	Neutralizes IL-25 to limit type 2 signaling	Phase I tolerability: subgroup AHR improvement; reduces total IgE and shows potential in type 2 inflammatory diseases
AEC-related	IL-17RB	SM17	Blocks IL-25 receptor signaling and downstream type 2 responses	Phase I tolerability in healthy volunteers; Reduces inflammation in HDM-challenged mice
Macrophage-related	Macrophage efferocytosis	Resolvin D2	Promotes clearance of apoptotic granulocytes and resolution of inflammation	Preclinical: enhances macrophage efferocytosis and partially restores inflammation resolution in particle-exposed allergic mice
Macrophage-related	NLRP3	RRx-001	Inhibits NLRP3 inflammasome activity and downstream IL-1β maturation	Preclinical in asthma: reduces airway inflammation and mucus production in HDM-challenged mice
Macrophage-related	C3 mRNA	miR-511-3p	Delivers mannose-decorated EV–miR-511-3p to lung macrophages and suppresses C3 expression	Preclinical: EV delivery reduces C3 expression and allergic airway inflammation in mice
Macrophage-related	SIRT6	Astragalin	Inhibits the SIRT6–LDHA–lactate–CXCL1/2 pathway that recruits neutrophils	Preclinical: SIRT6 inhibition reduces airway neutrophilia in asthmatic mice
Macrophage-related	PPAR-γ	Rosiglitazone	Activates macrophage lipid-metabolic programs and limits senescence-associated inflammation	Small randomized trials: limited clinical effects in asthma. Preclinical studies: reduces macrophage senescence and allergic airway inflammation in mice
Crosstalk-related	Epithelial circMETTL9	Aerobic exercise/circMETTL9 knockdown	Reduces circMETTL9-dependent CXCL12/CXCR4 signaling and MET formation	Preclinical: circMETTL9 overexpression counteracted exercise-induced MET suppression
Negative translation	IL-17RA	Brodalumab	Blocks IL-17 receptor signaling and is tested to reduce airway inflammation.	Randomized trial: no overall improvement in asthma control in moderate-to-severe asthma
Negative translation	IL-23p19	Risankizumab	Blocks IL-23 signaling and is tested as an anti-inflammatory strategy	Phase IIa: shorter time to asthma worsening versus placebo in severe asthma

This table summarizes therapeutic strategies targeting AECs, lung macrophages, and AEC–macrophage crosstalk in asthma, including their targets, interventions, mechanisms, and current clinical or experimental evidence.

AHR, airway hyperresponsiveness; FEV_1_, forced expiratory volume in one second; HDM, house dust mite.

### 7.1 Targeting AECs

Among strategies targeting AEC-derived mediators in asthma, TSLP neutralization has the strongest clinical validation. Tezepelumab, a monoclonal antibody that blocks TSLP, reduces exacerbations and improves lung function, asthma control, and health-related quality of life across type 2-high and type 2-low asthma populations (196, 197). In the CASCADE trial, tezepelumab reduced airway submucosal eosinophils and mannitol-induced AHR, but did not significantly improve epithelial integrity or reticular basement membrane thickness (198). Clinical efficacy of IL-33/ST2 blockade has been less consistent. Monoclonal antibodies targeting the IL-33/ST2 pathway include astegolimab, itepekimab, and tozorakimab. Astegolimab reduced exacerbation rates in severe asthma (199). Itepekimab improved asthma control, lung function, and quality of life in a phase II trial (200). Tozorakimab did not significantly improve the primary pre-bronchodilator FEV_1_ endpoint, although improvements in lung function were observed in patients with a greater history of exacerbations (201). Potential strategies targeting the IL-25 pathway include XKH001 (anti-IL-25 antibody) and SM17 (anti-IL-17RB antibody); however, their development for asthma remains at an early stage, with mechanistic appeal but clinical underdevelopment (202, 203).

### 7.2 Targeting lung macrophages

Therapeutic strategies targeting macrophages should preserve their homeostasis. AM depletion exacerbated IL-13- and HDM-induced type 2 inflammation and delayed inflammatory resolution in mice (204). Macrophage efferocytosis can restrain allergic inflammation, while resolvin D2 enhances efferocytosis and partially restores resolution impaired by ultrafine particles (205, 206). Conversely, inhibition of the macrophage NLRP3–caspase-1–IL-1β axis with RRx-001 reduced inflammatory cell infiltration and mucus production in HDM-induced allergic asthma (207). These findings support selective macrophage reprogramming or restoration of protective functions rather than indiscriminate depletion.

Macrophage immunometabolism represents an additional therapeutic target (111). Airway macrophage glycolysis can restrain type 2 immunity (208). In neutrophilic asthma, however, Su et al. found that sirtuin 6 (SIRT6) mediated macrophage immunometabolic reprogramming, subsequently driving neutrophilic asthma through lactate dehydrogenase A (LDHA)-dependent glycolysis, while treatment with astragalin (an SIRT6 inhibitor) reduced airway neutrophilia in mice (209). Macrophage-lineage PPAR-γ deficiency promoted macrophage senescence and allergic inflammation by disrupting lipid metabolic pathways, whereas macrophage-targeted treatment with rosiglitazone (a PPAR-γ agonist) attenuated these effects in mice (210); however, a randomized controlled trial of oral rosiglitazone showed modest attenuation of late asthmatic responses and limited lung-function improvement (211). In cockroach-allergen-challenged miR-511-3p-deficient mice, intratracheal delivery of mannose-decorated EV nanoparticles with miR-511-3p to lung macrophages reduced eosinophilic inflammation, goblet-cell hyperplasia, and type 2 cytokines. Mechanistic experiments identified C3 mRNA as a direct target, and treatment shifted macrophages toward more M2(F4/80+CD11c+Arg-1+) and fewer M1(F4/80+CD11c+iNOS+) phenotypes (212).

### 7.3 Targeting AEC–macrophage crosstalk

Direct modulation of AEC–lung macrophage communication represents a potential therapeutic strategy. Aerobic exercise downregulated epithelial circMETTL9 and reduced MET formation and allergic airway inflammation in mice; mechanistically, epithelial circMETTL9 is implicated in MET formation through CXCL12/CXCR4 signaling by competing with insulin-like growth factor binding protein 3 (IGFBP3) mRNA for the DEXDc domain of eukaryotic translation initiation factor 4A3 (EIF4A3) (213). The pathways discussed above also suggest potential strategies to interrupt pathogenic AEC–macrophage signaling or restore protective communication. Representative approaches include limiting CCL2/CCR2-mediated monocyte recruitment, targeting macrophage-derived CD30L or OSM signaling to epithelial cells, and supplementing protective SOCS3 delivery (91, 131, 153, 157, 168). These findings support further preclinical evaluation, with attention to the inflammatory context and the preservation of homeostatic function, although their relevance to human asthma remains unproven.

However, in a randomized phase II trial involving 302 patients with inadequately controlled moderate-to-severe asthma, IL-17RA blockade with brodalumab failed to improve asthma control (214). In another phase IIa trial involving 214 adults with severe asthma, IL-23p19 blockade with risankizumab also provided no clinical benefit and was associated with earlier and more frequent asthma worsening (215). These findings emphasize that mechanistic involvement does not necessarily translate into therapeutic efficacy and highlight the need for endotype-specific patient selection and human validation.

## 8 Conclusion and perspectives

AEC–lung macrophage crosstalk can promote inflammatory and remodeling responses in asthma or preserve airway homeostasis. AEC-derived alarmins, chemokines, cytokines, surface molecules, and EVs regulate macrophage recruitment and functional states, while macrophage-derived mediators, metabolites, extracellular traps, and EVs influence epithelial barrier function, mucus production, inflammation, and remodeling. The conceptual contribution of this review is to integrate these communication directions with asthma endotypes and the strength of the evidence, linking mechanistic interpretation to therapeutic relevance. This review highlights that a pathway’s effects may differ across experimental settings and that current evidence of bidirectional signaling does not, by itself, establish a self-sustaining feedback loop. These interactions also operate within broader multicellular networks.

The key knowledge gap is whether specific endogenous interactions between defined AEC and lung macrophage populations are necessary for disease persistence in human asthma, and how their contribution differs across asthma endotypes. Most causal and necessity evidence derives from experimental models. Human associations and functional responses in disease cohorts, or in isolated cells, support relevance but generally leave the endogenous source–recipient relationship incompletely resolved. Human allergen-challenge studies and single-cell and spatial analyses provide a basis for identifying disease-associated cellular states and candidate interactions (216–218).

Future work should connect these observations to complementary perturbations of the proposed source and recipient in primary-cell cocultures, organoid systems, and cell-specific *in vivo* models. Longitudinal sampling could further establish when candidate interactions emerge during exacerbation, resolution, or treatment. Extending these strategies to broader immune and structural-cell networks will also be essential for identifying clinically relevant multicellular circuits and biomarkers.

Therapeutic translation requires the same distinction between mediator activity and intercellular mechanism. Clinical efficacy of TSLP blockade does not establish that its benefits depend on AEC–macrophage signaling, while macrophage-directed and crosstalk-focused interventions remain largely preclinical. Ultimately, therapeutic strategies should be tailored to asthma endotypes, cell states, and disease stages. Prioritizing interactions with functional evidence in defined asthma endotypes may help identify interventions that suppress pathogenic signaling while preserving host defense, epithelial repair, and inflammatory resolution.
